# Perceptions of Healthy Aging Among Community-Dwelling Older Persons in an Urban Slum in Ibadan, Nigeria

**DOI:** 10.1093/geroni/igab046.1407

**Published:** 2021-12-17

**Authors:** Olubukola Omobowale

**Affiliations:** University of Ibadan, Nigeria, Ibadan, Oyo, Nigeria

## Abstract

Background: Healthy Ageing is the process of the development and maintenance of functional capacity which allows well-being at an older age. This understanding is comprehensive and relevant for all older persons. Despite the global attention being given to healthy ageing, there is limited information on the perceptions of this concept among older persons in low resource settings like Nigeria, hence the need for this study. Methods: In depth interviews were conducted among older persons aged 60 years and above residing in Idikan Community, an urban slum area in Ibadan, Nigeria. Using an interview guide, perceptions of older persons on meanings of healthy ageing, factors related to healthy ageing and experience of ageing were explored. Results: A total number of 24 interviews were conducted. Slightly more than half of the respondents were females. The majority of the respondents were of the opinion that healthy ageing is about being “ strong” and able to move around, without being dependent on anyone for mobility and activities of daily living. In their opinion, healthy ageing is related to different health dimensions: biological (adoption of healthy habits and behaviors with self-responsibility, psychological (feelings of optimism and happiness), spiritual (faith and religiosity) and family and social support (healthy and well children, friends and family). Conclusion: Urban Community dwelling older persons’ perception of healthy ageing was positive and incorporating their opinions on healthy aging from the perspective of the older persons can support the activities of professionals who work with this population group.

